# H_2_S-mediated protein S-sulfhydration: a novel regulatory module in lipid metabolism

**DOI:** 10.3389/fcell.2026.1866744

**Published:** 2026-06-17

**Authors:** Mingchang Pang, Haoyang Chen, Shuofeng Li, Huayu Yang, Yilei Mao, Yongfa Huang

**Affiliations:** 1 Department of Liver Surgery, Peking Union Medical College Hospital, Chinese Academy of Medical Sciences & Peking Union Medical College, Beijing, China; 2 Eight-year MD Program, Chinese Academy of Medical Sciences and Peking Union Medical College, Beijing, China; 3 Liver Transplantation Center, National Clinical Research Center for Digestive Diseases, Beijing Friendship Hospital, Capital Medical University, Beijing, China; 4 Clinical Center for Pediatric Liver Transplantation, Capital Medical University, Beijing, China; 5 Beijing Key Laboratory of Organ Cultivation and Organ Protection in Transplantation, Beijing Friendship Hospital, Capital Medical University, Beijing, China; 6 State Key Laboratory of Digestive Health, Beijing, China; 7 Laboratory for Clinical Medicine, Capital Medical University, Beijing, China

**Keywords:** hydrogen sulfide, lipid homeostasis, lipid metabolic disorders, redox signaling, S-sulfhydration

## Abstract

H_2_S-mediated protein S-sulfhydration is an emerging post-translational modification that regulates key biological processes via regulating enzyme activities, controlling protein–protein interactions, and modulating signal transduction. Lipid metabolism represents an important target of S-sulfhydration–mediated regulation, which fine-tunes lipid metabolic networks including fatty acid turnover, triglyceride metabolism, and cholesterol homeostasis. This review aims to systematically summarize current knowledge on the regulation of lipid metabolism with a focus on S-sulfhydration, and highlight novel molecular targets identified in recent research. By integrating emerging evidence, we demonstrate how S-sulfhydration acts as a regulatory module linking redox signaling and lipid homeostasis, which may be leveraged therapeutically to treat lipid-associated disorders, either using H_2_S donors or sulfhydrated protein–targeted medications.

## 1 Introduction

Lipid metabolism is a tightly regulated process critical for maintaining cellular and systemic homeostasis. Disrupted lipid homeostasis is closely associated with metabolic disorders, including hyperlipidemia, fatty liver disease, and cardiovascular and cerebrovascular diseases (Cao et al., 2025). Post-translational modifications (PTMs) of amino acid residues in proteins include phosphorylation, ubiquitination, as well as metabolite-derived acylations such as S-palmitoylation, crotonylation, succinylation and lactylation. These modifications play crucial roles in regulating lipid metabolism and are implicated in lipid-associated disorders (Feng et al., 2024; Francisco et al., 2024; Raju and Sankaranarayanan, 2025). Therefore, elucidating the roles of protein modifications in disease progression may provide new insights into underlying mechanisms and potential therapeutic strategies.

S-sulfhydration, also known as S-sulfuration or S-persulfidation, is a type of PTM in which hydrogen sulfide (H_2_S) or reactive polysulfides covalently attach a persulfide group (–SSH) to specific cysteine residues of target proteins. Accumulating studies demonstrate that S-sulfhydration exerts regulatory functions in multiple organs, including the nervous system, bone, cardiovascular system, liver, and kidney, which modulates mitochondrial function, autophagy, redox homeostasis, and inflammatory responses (Liu et al., 2025). Various types of proteins, including channels and transporters, enzymes, transcription factors, and other signaling molecules, have been shown to be regulated via protein S-sulfhydration (Luo et al., 2023a).

The role of S-sulfhydration in various biological and pathological contexts has been increasingly recognized. Since its regulatory function in lipid metabolism has not yet been systematically reviewed, this review aims to summarize current knowledge on the role of H_2_S-mediated S-sulfhydration in lipid metabolic pathways, and to discuss its potential relevance to lipid-associated disorders.

## 2 Biological production of H_2_S

**FIGURE 1:**
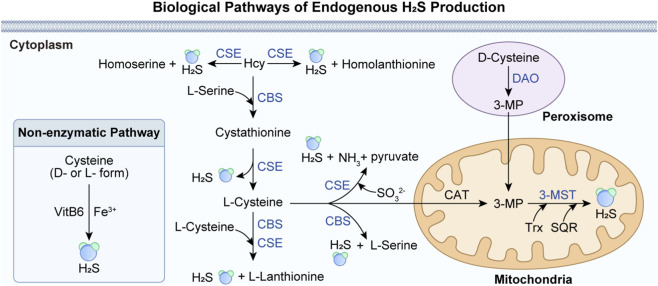
Endogenous H_2_S biosynthesis pathways. H_2_S is produced via cytoplasmic CBS/CSE-mediated transsulfuration, mitochondrial CAT/3-MST and DAO-3MST pathways, and non-enzymatic reactions.

H_2_S is recognized as the third endogenous gasotransmitter, in addition to nitric oxide and carbon monoxide. Endogenous H_2_S is primarily produced via cysteine-dependent pathways, with L-cysteine serving as the universal substrate for most enzymatic reactions. It can be generated via the transsulfuration pathway mediated by cystathionine-β-synthase (CBS) and cystathionine-γ-lyase (CSE). In addition, H_2_S is produced, as well as through the mitochondrial pathway involving cysteine aminotransferase (CAT) and 3-mercaptopyruvate sulfurtransferase (3-MST). In addition, H_2_S is produced through the mitochondrial pathway involving cysteine aminotransferase (CAT) and 3-mercaptopyruvate sulfurtransferase (3-MST). Besides L-cysteine, D-cysteine can contribute to H_2_S production via the D-amino acid oxidase (DAO)/3-MST pathway, while non-enzymatic reactions provide minor complementary contributions (Dilek et al., 2020) (Figure 1). Exogenous biologically produced H_2_S, on the other hand, is largely generated by gut microbiota.

### 2.1 CBS/CSE-mediated transsulfuration pathway

CBS and CSE are predominantly cytosolic enzymes that catalyze the metabolism of homocysteine (Hcy) and L-cysteine in a tissue-specific manner (Sun H. J. et al., 2021). CBS is highly expressed in the brain, liver, and kidney, with relatively lower expression detected in vascular tissues. In contrast, CSE primarily mediates H_2_S production in the liver, ileum, portal vein, thoracic aorta, and other non-vascular tissues (Lv et al., 2021).

Hcy–derived pathway: In this route, Hcy is first converted into cystathionine via a CBS-catalyzed reaction in the presence of L-serine, followed by CSE-mediated conversion of cystathionine into L-cysteine and H_2_S. Hcy can also serve directly as a substrate for CSE-dependent γ-elimination and γ-replacement reactions. These reactions produce H_2_S along with sulfur-containing by-products such as homoserine and homolanthionine.

L-cysteine–derived pathway: CBS catalyzes the hydrolysis of L-cysteine to generate H_2_S and L-serine in equimolar amounts. Two L-cysteine molecules can also undergo a β-replacement reaction in the presence of CBS and CSE to produce H_2_S and L-lanthionine. Additionally, CSE catalyzes the conversion of L-cysteine and sulfite (SO_3_^2-^) into L-cysteate and H_2_S, and also promotes the dimerization of L-cysteine to generate pyruvate and NH3, accompanied by H_2_S release (Han et al., 2019; Citi et al., 2018; Huang et al., 2023).

### 2.2 Mitochondrial CAT/3-MST pathway

In mitochondria, CAT and 3-MST constitute an independent H_2_S synthesis pathway. CAT converts L-cysteine into 3-mercaptopyruvate (3-MP), which is then metabolized by 3-MST to generate H_2_S, pyruvate, and persulfide intermediates. These persulfides are subsequently reduced by thioredoxin (Trx) or sulfide-quinone oxidoreductase (SQR) to release H_2_S (Dawoud et al., 2024).

### 2.3 DAO-3MST pathway

Another mitochondrial pathway, the DAO-3MST pathway, is highly enriched in the cerebellar tissues. In this way, D-cysteine, rather than L-cysteine, is specifically converted to 3-MP by DAO in peroxisomes, then transported to mitochondria, where 3-MST metabolizes it to produce H_2_S (Andrés et al., 2023; Zhong et al., 2020).

### 2.4 Non-enzymatic pathway

Non-enzymatic H_2_S production partially depends on cysteine, irrespective of its D- or L-isomeric form, and is catalyzed by vitamin B_6_ and ferric iron (Fe^3+^) (Yang et al., 2019). It occurs in multiple tissues including spleen, heart, lung, skeletal muscle, bone marrow, and plasma, with particularly high activity in red blood cells. Different vascular beds exhibit distinct H_2_S production capacities, reflecting tissue-specific regulation (Scammahorn et al., 2021).

### 2.5 Bacteria-derived H_2_S

In addition to host-derived enzymatic and non-enzymatic pathways, H_2_S is also abundantly produced by gut microbiota (Carbonero et al., 2012; Wolf et al., 2022; Birg and Lin, 2025). This occurs primarily via dissimilatory sulfate reduction by sulfate-reducing bacteria (SRB), and cysteine catabolism by diverse gut bacteria. Microbiota-derived H_2_S constitutes an important exogenous source that can reach high local concentrations in the intestinal lumen and diffuse across the gut barrier to influence host tissues (Wallace et al., 2018). Depending on concentration and pathological context, bacterial H_2_S may modulate protein S-sulfhydration and thereby impact host metabolic homeostasis (Lobel et al., 2020; Qi et al., 2024).

## 3 H_2_S-mediated protein S-Sulfhydration

S-sulfhydration is a recently identified post-translational modification induced by H_2_S or reactive polysulfides. It is defined as the covalent addition of–SSH to specific cysteine residues of target proteins. The selectivity of such reaction is determined by the pKa kinetics of local cysteine residues. Consequently, S-sulfhydration specifically modulates the activity of enzymes, alters subcellular structures by interfering protein–protein interactions, and regulates signaling transduction via transcription factor and other signaling proteins.

### 3.1 H_2_S-mediated S-sulfhydration in regulating enzymatic activity

S-sulfhydration modulates enzymatic activity by covalently modifying critical cysteine residues within catalytic or regulatory domains. CSE serves as a representative example: under physiological conditions, multiple cysteine residues (e.g., Cys84, Cys109, Cys229, Cys252 and Cys307) in CSE undergo S-sulfhydration, which is essential for maintaining its catalytic activity. Hyperhomocysteinemia augments CSE nitration, blunting its catalytic activity and causing H_2_S deficiency. The resulting reduction in H_2_S further diminishes CSE S-sulfhydration in a time-dependent manner, establishing a positive feedback loop of progressively impaired enzyme function. Site-directed mutagenesis confirms that S-sulfhydration at specific cysteine sites is required for optimal CSE enzymatic activity (Luo et al., 2021).

Likewise, H_2_S directly regulates mitochondrial ATP synthase activity by S-sulfhydrating Cys244 and Cys294 on the α-subunit (ATP5A1), thereby sustaining mitochondrial energy production. *In vitro*, NaHS induces ATP5A1 S-sulfhydration in a concentration-dependent manner, enhancing enzymatic activity. In contrast, mutation of both cysteines markedly diminishes ATP synthase activity. *In vivo*, CSE deficiency and reduced H_2_S levels decrease ATP5A1 S-sulfhydration and compromise enzymatic function, underscoring the critical role of S-sulfhydration in maintaining mitochondrial bioenergetics (Módis et al., 2016).

### 3.2 H_2_S-mediated S-sulfhydration in regulating protein–protein interactions

H_2_S-mediated S-sulfhydration dynamically regulates critical protein-protein interactions across diverse cellular contexts, including cytoskeletal signaling and mitochondrial dynamics. S-sulfhydration of β3 integrin modulates intramolecular disulfide bond formation, maintaining its extended, ligand-binding conformation. Loss of S-sulfhydration disrupts the interaction between β3 integrin and guanine nucleotide-binding protein subunit α13, leading to constitutive activation of Ras homolog family member A and impaired flow-induced endothelial cell realignment (Bibli et al., 2021).

Similarly, H_2_S directly regulates dynamin related protein 1 (Drp1) activity and protein–protein interactions by S-sulfhydration at Cys607. This modification competes with S-nitrosylation on Drp1 and suppresses Drp1–VDAC1 interactions. As a result, mitochondrial function and cellular viability are preserved. Deficiency of S-sulfhydration on Cys607 leads to Drp1 hyperactivation, excessive mitochondrial fission, and compromised cellular function, highlighting S-sulfhydration as a key mechanism regulating protein–protein interactions essential for cellular protection (Wu et al., 2022a).

### 3.3 H_2_S-mediated S-sulfhydration in regulating transcription factor activity

S-sulfhydration modulates the DNA-binding and transcriptional activity of transcription factors by altering the redox state of critical cysteine residues. For example, Hcy accumulation markedly reduces Sp1 S-sulfhydration, decreasing its binding to the promoter of CSE-encoding gene by approximately 40%, with the expression and subcellular localization of Sp1 unimpaired^18^. Moreover, H_2_S has been shown to suppress MMP2 transcription in vascular cells, at least in part via S-sulfhydration of Sp1. This modification selectively reduces transcriptional activity without affecting DNA binding. These findings suggest that S-sulfhydration functions as a redox-sensitive switch, fine-tuning the activity of Sp1 and potentially other transcription factors, thereby contributing to cellular protection and maintenance of tissue integrity, as demonstrated in aortic aneurysm and age-related medial degeneration (Zhu et al., 2022).

Endogenous CSE-derived H_2_S similarly promotes osteoblast differentiation, maturation, and mineralization via S-sulfhydration of transcription factor RUNX2. Specially, H_2_S modifies RUNX2 at cysteine residues C123 and C132, enhancing nuclear accumulation and binding to the OCN promoter. S-sulfhydration of RUNX2 upregulates osteogenic genes including ALP, collagen α1(I), and OCN. Loss of this persulfide modification or mutation of these cysteines abolishes H_2_S-dependent RUNX2 activation, highlighting a critical mechanism by which H_2_S fine-tunes transcription factor function during osteogenesis (Zheng et al., 2017).

## 4 Detection and profiling of protein S-Sulfhydration

Accurate detection of protein S-sulfhydration, characterized by the formation of cysteine persulfides (–SSH), remains technically challenging due to the chemical similarity between persulfides, thiols, and other reversible cysteine modifications. Recent methodological advances have substantially improved the specificity, sensitivity, and quantitative resolution of S-sulfhydration detection.

The most widely used approach is the modified biotin switch assay, which was adapted from S-nitrosylation detection. In this method, free thiols are first blocked by alkylating agents such as methyl methanethiosulfonate (MMTS), followed by selective reduction of persulfides and labeling with biotin-conjugated reagents (Módis et al., 2016). Biotinylated proteins can then be enriched by streptavidin pull-down and analyzed by immunoblotting or mass spectrometry, which serves as a proxy for protein S-sulfhydration levels. Notably, relatively high basal detection rates (up to ∼25% depending on the system and method) have raised concerns regarding potential methodological artifacts and false-positive signals (Pan and Carroll, 2013). Importantly, a major limitation of early switch-based approaches is their limited ability to distinguish S-sulfhydration from other reversible cysteine modifications, particularly S-nitrosylation. Because both persulfides and S-nitrosothiols can be reduced under similar experimental conditions, incomplete blocking or non-selective reduction may lead to cross-reactivity and overestimation of S-sulfhydration signals (Shi and Carroll, 2020). This specificity issue has been increasingly recognized as a key source of variability and inconsistency among published datasets.

To overcome these limitations, tag-switch assays were introduced to achieve higher specificity for persulfides detection. These methods exploit the distinct chemical reactivity of persulfides, which contain an additional sulfur atom with enhanced nucleophilicity compared with thiols. In tag-switch workflows, both thiols and persulfides are initially alkylated; however, only persulfide-derived alkylation products form reactive disulfide intermediates susceptible to subsequent nucleophilic substitution, whereas alkylated thiols remain inert (Shi and Carroll, 2020; Zhang et al., 2014; Yang et al., 2016). This design allows selective “switching” of persulfide signals while minimizing interference from conventional thiol modifications, thereby reducing cross-reactivity and false-positive detection. As a result, tag-switch approaches provide improved specificity for the detection and proteome-wide profiling of protein S-sulfhydration.

Persulfide-reactive fluorescent probes have been developed as convenient tools for monitoring protein S-sulfhydration in living cells (Zeng et al., 2015). Among these, the SSP-series probes are based on the selective reaction of persulfide species with thiol-containing probe scaffolds, leading to fluorescence activation. These probes enable real-time visualization of local persulfidation activity in response to metabolic or oxidative stimuli, thereby providing valuable spatial and temporal information (Shimamoto and Hanaoka, 2015; Takano et al., 2017). However, the readouts obtained from such fluorescent probes are largely qualitative and do not allow direct identification of modified proteins or modification sites. Therefore, complementary biochemical or proteomic approaches are required for molecular validation.

More recently, mass spectrometry–based proteomic strategies have been established for global and site-specific mapping of S-sulfhydrated cysteine residues. These approaches typically combine selective enrichment strategies with enzymatic digestion and MS/MS-based database searching to identify modified peptides and precisely localize cysteine modification sites (Shi and Carroll, 2020; Meng et al., 2018). These methods provide high-resolution and site-specific information on cysteine redox regulation but require stringent control of sample preparation, as incomplete blocking or processing artifacts may still introduce false-positive signals.

Collectively, no single method is sufficient to fully characterize protein S-sulfhydration. Integrated application of biochemical labeling, imaging probes, genetic validation (e.g., cysteine mutagenesis), and proteomic analysis is therefore recommended to reliably define S-sulfhydration events and their functional consequences in physiological and pathological contexts.

## 5 Lipid metabolism

**FIGURE 2:**
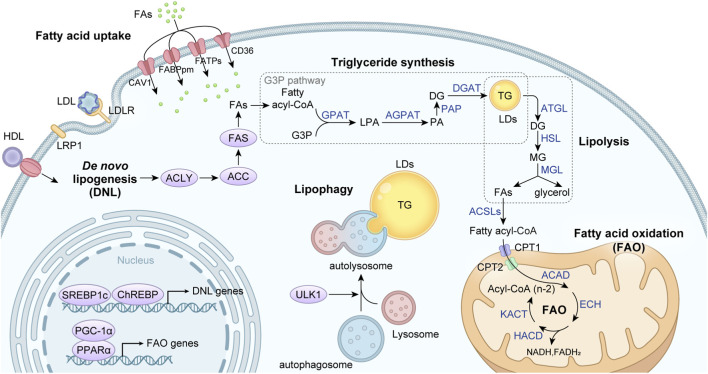
Overview of lipid metabolism, using fatty acid as a thread. Lipid metabolism is a highly coordinated network that mainly includes fatty acid uptake and *de novo* lipogenesis, fatty acid β-oxidation, triglyceride synthesis and catabolism, and cholesterol homeostasis.

Lipid metabolism is a highly regulated network encompassing fatty acid uptake and synthesis, fatty acid oxidation, triglyceride turnover, and cholesterol homeostasis, which is essential for metabolic stability at both cellular and organismal levels (Figure 2).

### 5.1 Fatty acid uptake and *de novo* lipogenesis

Fatty acids (FAs) are hydrophobic molecules that play diverse and essential roles in cellular physiology. FAs uptake refers to the transport of FAs across the plasma membrane and represents a critical step in cellular FA metabolism (Wang J. et al., 2025). Cells with high FA uptake capacity primarily include adipocytes, cardiomyocytes, hepatocytes, and endothelial cells. In most cell types, FA uptake is mediated by specific transport proteins, including CD36, fatty acid transport proteins (FATPs), plasma membrane fatty acid-binding protein (FABPpm), and caveolin-1 (CAV1) (Samovski et al., 2023; Acharya et al., 2023). Among these, CD36 is the predominant and most extensively studied mediator (Glatz et al., 2022).

*De novo* lipogenesis (DNL), another major source of FAs, refers to the process by which cells synthesize FAs from acetyl-CoA (Batchuluun et al., 2022; Lodhi et al., 2011). This process is primarily regulated at the transcriptional level by the transcription factors sterol regulatory element-binding protein 1 (SREBP1) and carbohydrate response element-binding protein (ChREBP). These transcription factors control the expression of genes involved in FA biosynthesis, including citrate/isocitrate carrier (CIC), ATP-citrate lyase (ACLY), acetyl-CoA carboxylase (ACC), and fatty acid synthase (FAS) (Wu et al., 2022b; Lane et al., 2019). Chronic upregulation of DNL is associated with cardiovascular disease, nonalcoholic fatty liver disease (NAFLD), and type 2 diabetes (Lai et al., 2019; Lawitz et al., 2018; Smith et al., 2020; Imamura et al., 2020).

### 5.2 Fatty acid catabolism

Fatty acid oxidation (FAO), principally β-oxidation, is the primary pathway for FA catabolism and a multi-step metabolic process that mainly occurs in mitochondria (Rashed et al., 2025). In the cytoplasm, FAs are first activated by fatty acyl-CoA synthetases (ACSLs) and then transported into mitochondria via the carnitine shuttle system. This involves conversion of acyl-CoA to acyl-carnitine by carnitine palmitoyltransferase 1 (CPT1) at the outer mitochondrial membrane, translocation across the inner membrane by carnitine-acylcarnitine translocase, and regeneration of acyl-CoA by carnitine palmitoyltransferase 2 (CPT2) within the mitochondrial matrix (Jiang et al., 2022; Selen et al., 2021). Once inside the matrix, fatty acyl-CoA undergoes progressive degradation. Each cycle produces acetyl-CoA and generates the energy carriers FADH_2_ and NADH to drive ATP synthesis. This process is catalyzed by a series of key enzymes: acyl-CoA dehydrogenase (ACAD), enoyl-CoA hydratase (ECH), 3-hydroxyacyl-CoA dehydrogenase (HACD), and ketoacyl-CoA thiolase (KACT) (Lopaschuk et al., 2010). Notably, mitochondrial β-oxidation represents the main pathway for metabolizing long-chain (C14-20) FAs, in which very-long-chain acyl-CoA dehydrogenase (VLCAD) catalyzes the initial dehydrogenation of acyl-CoA (Escudero et al., 2018; Wright et al., 2024). Deficiency of VLCAD has been shown to disrupt mitochondrial long-chain fatty acid β-oxidation (Tucci, 2017). The enzymes involved in FAO are tightly regulated at the transcriptional level, predominantly through peroxisome proliferator-activated receptor α (PPARα) and its coactivator, peroxisome proliferator-activated receptor γ coactivator-1α (PGC-1α) (Araújo et al., 2020; Li et al., 2015; Zhou et al., 2024).

### 5.3 Triglyceride synthesis and lipolysis

Triglyceride (TG) is the main form of energy storage in eukaryotic cells, predominantly sequestered within specialized organelles known as lipid droplets (LDs) (Xue et al., 2017). The liver, adipose tissue, and small intestine constitute the principal sites of TG synthesis, with the liver exhibiting the greatest synthetic capacity. TG biosynthesis relies on FAs and glycerol as fundamental substrates. In most mammalian cell types, the glycerol-3-phosphate (G3P) pathway accounts for more than 90% of total TG production. This process involves the stepwise esterification of fatty-acyl chains to G3P, initially catalyzed by glycerol-3-phosphate acyltransferases (GPATs), followed by acylglycerol-phosphate acyltransferases (AGPATs) to generate phosphatidic acid (PA). PA is subsequently dephosphorylated to diacylglycerol (DG) by lipin family phosphatidate phosphohydrolase (PAP), and the final acylation step catalyzed by diacylglycerol acyltransferases (DGATs) results in the formation of TG (Yu et al., 2025).

Lipolysis refers to the breakdown of TG stored in LDs (Zechner et al., 2012). The neutral hydrolysis of TG into FAs and glycerol occurs through three sequential enzymatic steps and requires at least three distinct lipases. Adipose triglyceride lipase (ATGL) catalyzes the initial and rate-limiting step, converting TG into DG. Hormone-sensitive lipase (HSL) predominantly mediates the hydrolysis of DG to monoacylglycerol (MG). Finally, monoacylglycerol lipase (MGL) completes the process by hydrolyzing MG to release free fatty acid (FFA) and glycerol (Grabner et al., 2021).

Besides lipolysis, lipophagy serves as an essential pathway for LDs catabolism (Zhang et al., 2025). During this process, LDs are engulfed by autophagosomes and subsequently delivered to lysosomes for degradation by lysosomal acid lipase (LAL). Autophagy is orchestrated by multiprotein complexes encoded by autophagy-related genes (ATGs), together with additional regulatory proteins (Nguyen et al., 2021a). The initiation of autophagy occurs when unc-51-like autophagy-activating kinase 1 (ULK1), a serine/threonine kinase, assembles into a complex with ATG13, focal adhesion kinase family–interacting protein of 200 kDa (FIP200), and ATG101, resulting in enhanced ULK1 kinase activity and protein stability. Beyond its role in autophagy initiation, ULK1 also regulates later stages of the process by interacting with the soluble N-ethylmaleimide–sensitive factor attachment protein receptor protein syntaxin 17, thereby influencing autophagosome–lysosome fusion (Ro et al., 2013; Nguyen et al., 2021b; Wang et al., 2018).

### 5.4 Cholesterol metabolism

Cholesterol homeostasis is maintained through coordinated processes including synthesis, absorption, transport, conversion, and clearance. The liver serves as the central regulatory organ of the process. *De novo* cholesterol synthesis occurs mainly in the liver, with minor contributions from extrahepatic tissues. Acetyl-CoA is the carbon source, and ATP and NADPH are consumed. In addition to endogenous synthesis, cholesterol is absorbed from the intestine, primarily in the duodenum and proximal jejunum, via the enterocyte membrane protein Niemann-Pick C1-like 1. Internalized cholesterol is either esterified by acyl-CoA:cholesterol acyltransferase two and incorporated into chylomicrons for transport to the liver, or effluxed back into the intestinal lumen by ATP-binding cassette transporters G5/G8 (Luo et al., 2020; Duan et al., 2022).

Within the liver, cholesterol can be packaged into very-low-density lipoproteins (VLDL) and secreted into the circulation. During circulation, VLDL is progressively remodeled into intermediate-density lipoproteins (IDL) and low-density lipoproteins (LDL), which deliver cholesterol to extrahepatic tissues (Vaziri, 2016). Excess circulating LDL is subsequently cleared by the liver through low-density lipoprotein receptor (LDLR)-mediated endocytosis. LDL particles carrying cholesterol esters (CE) and apolipoprotein B-100 (ApoB-100) bind LDLR on hepatocytes and are internalized into lysosomes, where CE are hydrolyzed to free cholesterol. In addition, hepatic low-density lipoprotein receptor–related protein 1 (LRP1) cooperates with LDLR to mediate the clearance of cholesterol-rich chylomicron remnants from the circulation (Mineo, 2020; Actis Dato and Chiabrando, 2018).

Complementing this pathway, high-density lipoprotein (HDL) mediates reverse cholesterol transport (RCT), returning excess cholesterol from peripheral tissues to the liver for disposal. Ultimately, hepatic cholesterol can be converted into bile acids by cholesterol 7α-hydroxylase or oxidized to oxysterols, which function as signaling molecules regulating metabolic pathways (Groen et al., 2014; Schroepfer, 2000; Poli et al., 2022).

## 6 Emerging roles of H_2_S and protein S-sulfhydration in lipid metabolic homeostasis

**FIGURE 3:**
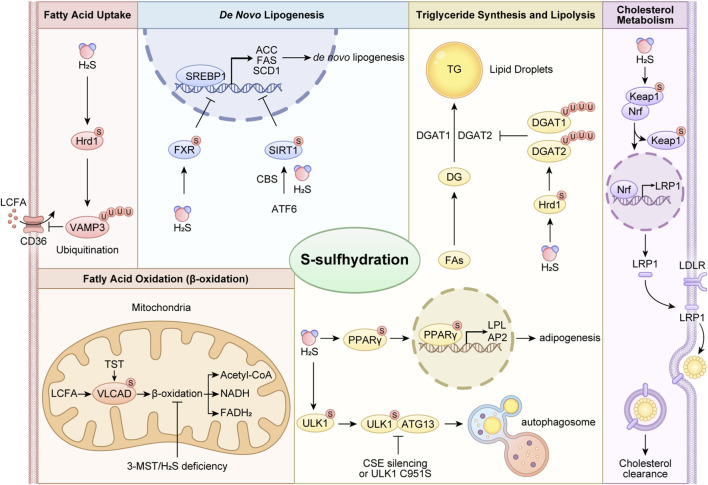
Mechanistic framework of H_2_S-mediated protein S-sulfhydration in lipid metabolic homeostasis. S-sulfhydration regulates lipid metabolism by modulating enzyme activity, remodeling protein–protein interactions, and controlling transcription factors, thereby integrating redox signaling with lipid metabolic networks.

Accumulating evidence suggests that H_2_S-mediated protein S-sulfhydration regulates lipid metabolism through three principal functional modes: directly modulating the activity of enzymes, dynamically controlling protein-protein interactions, and modifying transcriptional factors and other signaling proteins within metabolic regulatory networks (Figure 3; Table 1).

**TABLE 1:** S-sulfhydration-mediated regulation of lipid metabolism.

Target protein	Experimental model	S-sulfhydrated cysteine residues	Effects of S-sulfhydration	Functional outcomes	References
Hrd1	db/db mice; NRCMs	Cys115	Inhibition of CD36-mediated fatty acid uptake	↓ FA uptake ↓ TG	Yu et al. (2020)
SIRT1	ATF6-cKO mice	NA	Inhibition of SREBP1-mediated lipogenesis	↓ FA de novo synthesis; anti-inflammatory effects	Dong et al. (2023)
Hrd1	db/db mice HL-1 cells	Cys115	Suppression of SREBP1-mediated lipogenesis	↓ TG	Zhang et al. (2023)
FXR	CSE^LKO^ mice	Cys138 Cys141	Repression of *de novo* lipogenesis	↓ FA de novo synthesis ↓ Insulin resistance ↓ Hepatic fibrosis	Xu et al. (2022)
VLCAD	TST-KO mice; HK-2 cells	Cys237	Enhancement of VLCAD-dependent mitochondrial FAO	↑ FAO	Zhang J. X. et al. (2024)
Hrd1	db/db mice	Cys115	Suppression of DGAT1/2-dependent triglyceride synthesis	↓Lipid droplets	Sun Y. et al. (2021)
PPARγ	CSE-KO mice 3T3-L1 cells	Cys148, Cys152, Cys162, Cys165 Cys170	Stimulation of triglyceride accumulation in adipocytes	↑ adipogenesis; ↑ TG	Yang et al. (2018)
ULK1	HFD-fed mice	Cys951	Promotion of ULK1-dependent lipophagy	↑ lipophagy ↓ TG	Nguyen et al. (2021b)
Keap1	*Ldlr* ^−/−^ mice CCl_4_-treated mice	Cys151	Promotion of Nrf2–LRP1–mediated hepatic cholesterol clearance	↑ cholesterol clearance ↓ hepatic lipid accumulation	Zhao et al. (2021)

### 6.1 Protein S-sulfhydration in fatty acid uptake

The predominant FA transporter CD36 dynamically translocates from an intracellular pool to plasma membrane in response to metabolic stimuli. Using both type 2 diabetic db/db mice and neonatal rat cardiomyocytes (NRCMs), it was demonstrated that S-sulfhydration of the E3 ubiquitin ligase Hrd1 at Cys115 enhanced the ubiquitination and degradation of VAMP3, a vesicle-associated membrane protein. This process limits VAMP3-mediated translocation of CD36 to the plasma membrane, resulting in reduced FAs uptake and attenuated intracellular TG accumulation in cardiomyocytes (Yu et al., 2020). The Hrd1–VAMP3–CD36 axis thus represents a redox-dependent mechanism linking S-sulfhydration to vesicle trafficking and lipid uptake in diabetic cardiomyopathy. As VAMP3 participates in general vesicular trafficking processes, its regulation by S-sulfhydration may exert broader effects on the membrane localization of lipid transport proteins beyond CD36.

### 6.2 Protein S-sulfhydration in *de novo* lipogenesis

SREBP1 is a master transcription factor controlling cardiac and hepatic lipogenesis. In diabetic hearts, SREBP1 exhibits enhanced translocation from the endoplasmic reticulum to the nucleus, thereby driving FAs synthesis and LDs accumulation. H_2_S counteracts this process by inducing S-sulfhydration of Hrd1 at Cys115, which promotes ubiquitination and proteasomal degradation of SREBP1, ultimately limiting its nuclear accumulation and transcriptional activity. Consistently, treatment with H_2_S donors such as NaHS or GYY4137 in db/db mice restores Hrd1 expression and sulfhydration, reduces SREBP1 and DGAT1 levels, and markedly attenuates TG deposition (Zhang et al., 2023). In the liver, SREBP1-driven lipogenesis is further modulated by protein S-sulfhydration through regulatory factors such as sirtuin (SIRT1) and CBS/H_2_S axis. In liver specific ATF6 knockout mice, GYY4137-mediated H_2_S supplementation restores SIRT1 S-sulfhydration, thereby inhibiting SREBP1-driven lipogenic gene expression and consequent suppression of FAs synthesis. Mechanistically, ATF6 normally enhances endogenous H_2_S production through transcriptional upregulation of CBS, leading to increased SIRT S-sulfhydration. Beyond metabolic regulation, sulfhydrated SIRT1 also exerts anti-inflammatory effects by downregulating the pro-inflammatory cytokine IL-17A while upregulating IL-10, thereby alleviating hepatic inflammation (Dong et al., 2023). Collectively, these findings indicate that H_2_S-dependent S-sulfhydration modulates *de novo* lipogenesis through coordinated control of transcription factor stability and inflammatory signaling, beyond isolated modulation of individual lipogenic enzymes.

CSE-derived H_2_S also restrains hepatic lipogenesis through direct S-sulfhydration of the nuclear receptor farnesoid X receptor (FXR) at Cys138 and Cys141, thereby enhancing its transcriptional activity to repress lipogenic gene pathways. In hepatocyte-specific CSE knockout mice, loss of H_2_S diminishes FXR S-sulfhydration, resulting in derepression of *de novo* lipogenesis and coordinated upregulation of canonical lipogenic regulators, including SREBP-1, ACC, FAS, and stearoyl-CoA desaturase 1 (SCD1). Notably, reduced hepatic H_2_S bioavailability observed in patients with NAFLD and in high-fat diet–fed mouse models correlates with excessive lipid accumulation, insulin resistance, and metabolic dysfunction (Xu et al., 2022). These findings define a CSE–H_2_S–FXR S-sulfhydration axis as a redox-sensitive checkpoint that links protein PTM to transcriptional control of FAs synthesis, thereby safeguarding hepatic lipid homeostasis.

Beyond modulation of downstream effectors such as Hrd1, SIRT1, and FXR, hepatic lipid homeostasis is also governed by dynamic expression of endogenous H_2_S-producing enzymes. Excessive FFA accumulation has been shown to induce 3-MST expression and suppress CSE expression in the liver of HFD–fed mice as well as in patients with NAFLD, which consequently impaired hepatic H_2_S synthesis. This disruption activates SREBP1-dependent lipogenic programs, enhances JNK phosphorylation and oxidative stress, and ultimately accelerates hepatic lipid accumulation. Genetic or pharmacological inhibition of 3-MST restores H_2_S signaling and suppresses lipogenesis, underscoring a pathogenic feedback loop linking lipid overload to sustained SREBP1 activity (Li et al., 2018). The association between upregulation of 3-MST and impairment of H_2_S signaling seemed paradoxical at the first glance. From a bird’s view, however, this work reminds us to treat the H_2_S production sources as an interconnected, self-balancing system. When determining the H_2_S producibility, all potential H_2_S producing enzymes, non-enzymatic reactions, as well as microbiota should be considered collectively.

### 6.3 H_2_S/protein S-sulfhydration in fatty acid oxidation

Protein S-sulfhydration and H_2_S signaling are emerging as key regulators of mitochondrial FAO. Impaired FAO in renal tubular cells is a hallmark of diabetic kidney disease (DKD) and contributes to tubular injury and tubulointerstitial fibrosis. Thiosulfate sulfurtransferase (TST), a mitochondrial enzyme downregulated in diabetes, utilizes thiosulfate—predominantly derived from mitochondrial H_2_S oxidation—to generate reactive polysulfides for S-sulfhydration and serves as a key regulator of tubular FAO (Luo et al., 2025; Kanemaru and Ichinose, 2025). Reduced TST expression diminishes S-sulfhydration of VLCAD, a rate-limiting enzyme in mitochondrial β-oxidation of long-chain FAs, leading to mitochondrial dysfunction and defective FAO and exacerbated renal fibrosis, as demonstrated in TST knockout mice, particularly under diabetic conditions. Furthermore, S-sulfhydration of VLCAD at Cys237 is essential for maintaining mitochondrial respiration, ATP production, and redox homeostasis under high-glucose conditions in HK-2 cells. While wild-type VLCAD preserves FAO activity and mitigates oxidative stress and fibrotic responses in renal tubular cells, S-sulfhydration–deficient VLCAD mutants fail to confer these protective effects (Zhang J. X. et al., 2024). Taken together, TST-mediated S-sulfhydration of VLCAD provides a mechanistic link between sulfur metabolism, mitochondrial FAO, and renal tubular protection in DKD.

Similarly, in adipose tissue, H_2_S also acts as an important metabolic regulator that restrains adipocyte differentiation and lipid accumulation. Pharmacological inhibition or genetic deletion of 3-MST enhances adipogenesis and lipid storage, whereas H_2_S administration suppresses these processes. 3-MST deficiency impairs mitochondrial respiration and FAO, as evidenced by reduced oxygen consumption, ATP production, and FAO capacity in adipose tissue of 3-MST knockout mice. The resulting decline in mitochondrial FAO shifts cellular metabolism toward lipid accumulation and promotes adipocyte maturation (Casili et al., 2022). Collectively, these results identify the 3-MST/H_2_S axis as a key mitochondrial regulator of FAO and adipocyte differentiation, suggesting that restoration of H_2_S signaling may represent a potential strategy to counteract age- and metabolism-associated adipose tissue dysfunction.

Likewise, in the liver, mitochondrial β-oxidation is central to FFA catabolism, and its impairment drives lipotoxicity under high-fat diet conditions. In hepatocytes from HFD-fed hamsters, the H_2_S donor anethole dithiolethione (ADT) enhances FAO by upregulating CPT1α and promoting mitochondrial fusion, facilitating FAs uptake, mitochondrial entry, and β-oxidation (Zhao et al., 2020). These results underscore the role of H_2_S in coordinating mitochondrial dynamics and β-oxidation to counteract lipid accumulation and lipotoxicity in hepatocytes, and the specific molecular processes require further investigation.

Overall, H_2_S-dependent S-sulfhydration coordinates mitochondrial fatty acid oxidation by regulating key enzymes, preserving mitochondrial function, and maintaining cellular energy and redox homeostasis across liver, adipose tissue, and kidney.

### 6.4 Protein of S-sulfhydration in triglyceride synthesis and lipolysis

H_2_S-dependent S-sulfhydration is an essential mechanism controlling TG formation and degradation. In db/db mice, exogenous H_2_S treatment reduced LDs accumulation and improved cardiac function. Mechanistically, H_2_S induces S-sulfhydration of Hrd1 at Cys115, which enhances its interaction with DGAT1 and DGAT2, key enzymes catalyzing TG formation. This interaction promotes the ubiquitination and proteasomal degradation of DGAT1 and DGAT2, leading to decreased TG synthesis and reduced LDs formation (Sun Y. et al., 2021). These findings demonstrate that protein S-sulfhydration of Hrd1 is a critical regulatory mechanism that prevents excessive lipid accumulation in cardiomyocytes.

Interestingly, while protein S-sulfhydration can inhibit TG synthesis in cardiac tissues, it also exerts a pro-adipogenic effect in adipocytes. Functionally, H_2_S S-sulfhydrates peroxisome proliferator-activated receptor γ (PPARγ) at Cys148, Cys152, Cys162, Cys165, Cys170 in the DNA-binding domain, enhancing its transactivation activity and promoting the expression of downstream target genes such as lipoprotein lipase (LPL) and adipocyte protein 2 (Ap2). *In vivo*, CSE deficiency reduces HFD induced fat mass in mice, whereas exogenous H_2_S promotes fat accumulation in model organisms (Yang et al., 2018). These findings indicate that the CSE/H_2_S system facilitates adipocyte differentiation and lipid storage through direct activation of PPARγ. Notably, although such adipogenic effects may be metabolically adaptive by promoting lipid accumulation and limiting ectopic lipotoxicity, sustained or excessive activation of this pathway under chronic overnutrition could conversely exacerbate adiposity and metabolic burden. Together, these observations highlight the context-dependent and potentially double-edged roles of protein S-sulfhydration in lipid metabolism.

In HFD–induced hepatic steatosis, activation of SREBP-1 upregulates miR-216a, which suppresses CSE expression and reduces H_2_S production. Decreased H_2_S levels impair the S-sulfhydration of ULK1 at Cys951, leading to inhibition of autophagic flux and LDs turnover. Specifically, S-sulfhydration of ULK1 enhances its intrinsic kinase activity and stabilizes the ULK1-ATG13-FIP200 complex during autophagy initiation, promoting phosphorylation of downstream targets such as Atg13 and Atg14, and facilitating autophagosome formation. Mutation of ULK1 at Cys951 or CSE silencing disrupts ULK1 S-sulfhydration, reduces kinase activity, inhibits autophagic flux, and increases association of UVRAG with Rubicon, thereby blocking autophagosome-lysosome fusion and inhibiting hepatic lipophagy (Nguyen et al., 2021b). Accordingly, ULK1 S-sulfhydration is required for both the initiation and progression of lipophagy, functionally linking H_2_S/S-sulfhydration signaling to hepatic lipid catabolism.

Taken together, protein S-sulfhydration acts as a central regulatory hub in triglyceride metabolism, coordinating enzyme activity, transcription factor function, and autophagic flux to fine-tune lipid storage and mobilization. Its effects are tissue-specific: it limits excessive TG accumulation in cardiomyocytes, promotes adipocyte differentiation and lipid storage, and modulates hepatic lipophagy to maintain lipid homeostasis.

### 6.5 Protein S-sulfhydration in cholesterol metabolism

Disruption of H_2_S-mediated protein S-sulfhydration has been closely linked to disordered hepatic cholesterol metabolism under metabolic stress. In streptozotocin (STZ)–treated and HFD–fed LDL receptor–deficient (*Ldlr* ^−/−^) mice, as well as in patients with fatty liver under diabetic conditions, impaired H_2_S signaling is associated with markedly reduced S-sulfhydration of Kelch-like ECH-associated protein 1 (Keap1), leading to suppression of nuclear erythroid 2–related factor 2 (Nrf2) activity. Consistently, in STZ + HFD–treated *Ldlr* ^−/−^ mice and in primary mouse hepatocytes exposed to high glucose and oxidized LDL, restoration of Keap1 S-sulfhydration by the slow-releasing H_2_S donor GYY4137 promotes Nrf2 dissociation from Keap1, facilitates its nuclear translocation, and enhances transcriptional activation of antioxidant and lipid-handling genes. Among these targets, LRP1 is directly upregulated through increased Nrf2 binding to its promoter, thereby cooperating with LDLR to mediate hepatic uptake and clearance of cholesterol-rich lipoprotein remnants. Importantly, mutation of Keap1 at Cys151, the critical S-sulfhydration site, abolishes hepatoprotective effects of H_2_S both *in vivo* and *in vitro*, while genetic deletion of Nrf2 similarly negates the beneficial impacts of H_2_S on cholesterol handling and liver injury. Parallel findings in CCl_4_-induced liver injury models further support a conserved role of Keap1 S-sulfhydration in activating Nrf2 signaling to alleviate oxidative stress, lipid accumulation, and hepatic damage (Zhao et al., 2021). Collectively, these findings establish H_2_S-mediated Keap1 S-sulfhydration as a redox-sensitive molecular switch that links metabolic stress to Nrf2-driven LRP1 expression and hepatic cholesterol homeostasis.

## 7 Tissue-specific roles of H_2_S-mediated protein S-sulfhydration in lipid metabolic dysregulation

Collectively, H_2_S-mediated protein S-sulfhydration represents an important regulatory module underlying lipid metabolic dysregulation across diverse diseases. Despite tissue-specific targets, a common pattern emerges in which impaired H_2_S bioavailability or defective S-sulfhydration disrupts lipid homeostasis and accelerates disease progression. In diabetic cardiomyopathy, impaired protein S-sulfhydration has been associated with overwhelming fatty acid uptake and lipogenesis, thereby exacerbating insulin resistance and cardiac dysfunction. In diabetic kidney disease and other chronic kidney diseases, loss of protein S-sulfhydration primarily disrupts FAO and produces excessive reactive oxygen species, resulting in tubular lipid accumulation and fibrotic remodeling. In NAFLD, diminished protein S-sulfhydration shifts hepatic lipid metabolism toward enhanced *de novo* lipogenesis and impaired lipid catabolism, thereby promoting hepatic steatosis and disease progression. In adipocytes, adequate H_2_S production and S-sulfhydration are required for adipogenesis and fat mass accumulation (Yang et al., 2018).

Defective S-sulfhydration appears more detrimental in the heart and the kidney compared with the liver case, which could potentially be attributed to organ-specific metabolic architectures. Lipid metabolism scenario in the heart and the kidney largely presents as energy-generating mitochondrial fatty acid oxidation, whose disruption directly hampers the energy-demanding physiological activities and leads to secondary damages from oxidative stress and lipotoxicity (Zhang J. X. et al., 2024; Wang M. et al., 2025; Zhang H. et al., 2024). In contrast, the liver, as well as adipose tissue, serves as a hub of systemic lipid metabolism, which physiologically buffers the fluctuation of lipid content within the plasma and other segments of the internal environment (Xu et al., 2022; Casili et al., 2022). Under dysregulated conditions, defective S-sulfhydration in the liver may not manifest as severe anomaly *in situ*, but the consequent systemic dyslipidemia should exert acute or chronic pathological changes in other tissues and organs. Such spatially distinct regulatory patterns underscore the importance of considering organ-specific contexts when interpreting the role of S-sulfhydration in metabolic homeostasis and disease progression.

## 8 Therapeutic targeting of H_2_S-mediated protein S-sulfhydration: donor strategies and translational barriers

Therapeutic strategies aimed at restoring H_2_S-mediated protein S-sulfhydration have attracted increasing attention as potential interventions for lipid metabolic disorders. Currently, exogenous H_2_S donors represent the most widely explored pharmacological tools, and these compounds differ substantially in their release kinetics, bioavailability, and safety profiles. Fast-releasing sulfide salts, such as NaHS, rapidly elevate systemic H_2_S levels and are therefore useful for acute experimental modulation of S-sulfhydration (Szabo and Papapetropoulos, 2017). Nevertheless, their tendency to induce supraphysiological H_2_S concentrations substantially limits translational applicability due to dose-dependent toxicity. In contrast, slow-releasing H_2_S donors, exemplified by GYY4137, provide a more sustained and physiologically relevant supply of H_2_S (Liu et al., 2025; Magli et al., 2021). Following oral or intraperitoneal administration, these compounds are absorbed into the circulation and distributed across tissues. Their metabolites are predominantly converted to thiosulfate and sulfate and subsequently excreted in the urine. This controlled release profile has been associated with anti-inflammatory, antioxidant, and anticancer (Luo et al., 2023b; Oberholtzer et al., 2024) effects in multiple experimental models, although metabolic disease–specific evidence remains limited. Furthermore, mitochondria-targeted H_2_S donors, including ADT-based compounds, offer an additional layer of spatial precision by preferentially enhancing local S-sulfhydration of mitochondrial metabolic enzymes, thereby improving therapeutic efficacy while minimizing off-target effects (Powell et al., 2018). To date, clinical evaluation of H_2_S donors remains limited to early-phase studies of compounds such as SG1002 and ATB-346, which have primarily assessed safety, pharmacokinetics, and anti-inflammatory or cardiovascular outcomes, rather than metabolic outcomes (Polhemus et al., 2015; Wallace et al., 2020; Glanville et al., 2021). Notably, no clinical studies have directly investigated H_2_S donors or protein S-sulfhydration in lipid metabolic disorders, underscoring a substantial translational gap between mechanistic preclinical evidence and disease-specific clinical application.

Despite promising preclinical findings, several challenges persist. The pharmacokinetic behavior of H_2_S donors remains incompletely characterized, with large inter-organ variability in H_2_S metabolism and rapid clearance complicating dose optimization. Moreover, unregulated H_2_S supplementation raises safety concerns, as excessive sulfide exposure may impair mitochondrial respiration and redox balance in non-target tissues (Liu et al., 2025). Importantly, most current evidence derives from short-term animal studies (Zhang et al., 2023; Sun Y. et al., 2021), and robust clinical data evaluating efficacy, toxicity, and long-term outcomes specifically in lipid metabolism–related diseases are lacking. Future therapeutic development may therefore benefit from alternative strategies, such as enhancing the activity of endogenous H_2_S-producing enzymes, designing targeted S-sulfhydration modulators, or developing tissue-specific delivery systems (Wallace and Wang, 2015). Collectively, these considerations highlight both the therapeutic potential and current limitations of targeting H_2_S-mediated S-sulfhydration in lipid metabolic diseases.

## 9 Critical evaluation of S-sulfhydration in lipid metabolism

Despite rapid advances in elucidating the role of H_2_S-mediated S-sulfhydration in lipid metabolism, several important limitations should be acknowledged. First, the majority of current evidence is derived from *in vitro* and animal models, whereas clinical validation in human metabolic diseases remains limited, making the translational relevance of many findings uncertain. Second, the specificity of S-sulfhydration detection remains a major methodological challenge. Commonly used approaches, including tag-switch chemistry and mass spectrometry–based enrichment, may be influenced by competing thiol modifications, redox state fluctuations, and incomplete site occupancy, which together complicate precise identification of physiologically relevant modification sites. Third, most studies establish associations between altered H_2_S levels, changes in S-sulfhydration, and lipid metabolic phenotypes, but direct evidence demonstrating causality remains far from satisfaction. Despite that methodologies including site-directed mutagenesis partially address Koch’s postulates, one fact remains confusing as in the cases of other PTMs: with all potential artifacts ruled out, there are still some proteins with increased S-sulfhydration upon decreased H_2_S levels, and *vice versa*. Finally, accumulating evidence indicates strong tissue-specific and context-dependent effects of S-sulfhydration, as it can either suppress lipid accumulation in cardiomyocytes, promote adipogenesis in adipose tissue, or regulate lipid turnover in the liver. These seemingly contradictory effects underscore the complexity of H_2_S signaling and support the view that S-sulfhydration operates as a context-dependent regulatory mechanism rather than a uniform metabolic switch.

## 10 Perspectives and future directions

Accumulating evidence summarized in this review establishes H_2_S-mediated protein S-sulfhydration as a critical redox regulatory mechanism governing lipid metabolic homeostasis, influencing fatty acid handling, triglyceride turnover, and cholesterol homeostasis across multiple tissues. Given that lipid metabolism is a highly coordinated and finely regulated process, perturbations in H_2_S bioavailability and aberrant S-sulfhydration can disrupt multiple metabolic nodes and contribute to the development and progression of lipid-associated disorders, including NAFLD, atherosclerosis, diabetic cardiomyopathy, and obesity-associated metabolic dysfunction.

In this context, despite recent advances, the tissue-specific nature of S-sulfhydration signaling remains poorly defined. Identical sulfhydration events may elicit divergent metabolic outcomes depending on cellular and pathological contexts, underscoring the necessity for spatially resolved and tissue-targeted investigations. Moreover, beyond its widely reported protective roles, the potential detrimental or maladaptive effects of S-sulfhydration remain insufficiently explored and warrant systematic evaluation. Importantly, future studies should move beyond isolated enzymatic reactions and instead focus on higher-order regulatory processes, such as lipoprotein assembly and secretion, where coordinated control of lipid packaging and export is pivotal for maintaining systemic lipid homeostasis.

From a translational perspective, H_2_S donors offer promising therapeutic potential for lipid metabolic diseases; however, challenges related to tissue specificity, controlled release, and long-term safety must be addressed before clinical application. Bacteria-derived exogenous H_2_S represents an important component of host sulfur metabolism, yet its role in regulating host lipid metabolism—particularly through protein S-sulfhydration—remains unexplored. Elucidating how microbiota-derived H_2_S influences systemic lipid homeostasis may uncover novel host–microbe regulatory axes and therapeutic opportunities.
